# Germination and Polishing Reshape Microbial Communities in *Japonica* and *Indica* Rice

**DOI:** 10.1021/acs.jafc.6c02819

**Published:** 2026-07-08

**Authors:** Maria Eugenia Araujo Silva Oliveira, Daniel Lucino, Glen Jasper Yupanqui Garcia, Bruno Gerfi Bertozzi, Priscila Zaczuk Bassinello, José Manoel Colombari Filho, Carlos Wanderlei Piler de Carvalho, Aristóteles Góes-Neto, Liliana de Oliveira Rocha, Dirce Yorika Kabuki, Otniel Freitas Silva, Cristina Yoshie Takeiti

**Affiliations:** † Programa de Pós-Graduação em Alimentos e Nutrição (PPGAN), Universidade Federal do Estado do Rio de Janeiro, UNIRIO, Avenida Pateurs, 296, 22290-240 Rio de Janeiro, Rio de Janeiro, Brasil; ‡ Departamento de Ciência de Alimentos e Nutrição (DECAN), Programa de Pós-Graduação em Ciência de Alimentos, Faculdade de Engenharia de Alimentos (FEA), 28132 Universidade Estadual de Campinas (UNICAMP), Rua Monteiro Lobato, 80, Cidade Universitária Zeferino Vaz, 13083-862 Campinas, São Paulo, Brasil; § Departamento de Microbiologia, Programa de Pós-Graduação em Bioinformática, Instituto de Ciências Biológicas, 28114 Universidade Federal de Minas Gerais, UFMG, Avenida Antônio Carlos, 6627, Pampulha, 31270-901 Belo Horizonte, Minas Gerais, Brasil; ∥ Embrapa Alimentos e Territórios, Rua Cincinato Pinto, 348, 57020-050 Maceió, Alagoas, Brasil; ⊥ Embrapa Arroz e Feijão, Rodovia GO-462, Km 12, Zona Rural, 75375-000 Santo Antônio de Goiás, Goiás, Brasil; # Embrapa Agroindústria de Alimentos, Avenida das Américas, 29501, 23020-470 Rio de Janeiro, Rio de Janeiro, Brasil

**Keywords:** germination, polishing, metagenomics, alpha-diversity, beta-diversity

## Abstract

Germination is a process used to improve the nutritional quality of rice. However, its impact on rice microbiomes remains poorly understood. This study evaluated the microbiota of two rice ecotypes, low-amylose (Mochi) and high-amylose (BRS Formoso), after germination and polishing using 16S rRNA and ITS amplicon sequencing. Bacterial alpha diversity was highest in commercial brown rice (Shannon index 3.21) and lowest in commercial polished rice (1.50). Beta diversity indicated that germination exerted a similar effect on bacterial community composition in both ecotypes. Principal Coordinate Analysis suggested that polishing did not markedly influence microbiome composition relative to germination. The microbial profiles of Mochi and BRS Formoso were dominated by *Pantoea*, *Pseudomonas*, *Rhizopus*, and *Moesziomyces*. Overall, germination strongly influenced bacterial and fungal communities, emerging as the main factor shaping microbial structure and dynamics. These findings provide new insights into how processing affects the rice microbiome, with implications for food quality and safety.

## Graphical abstract

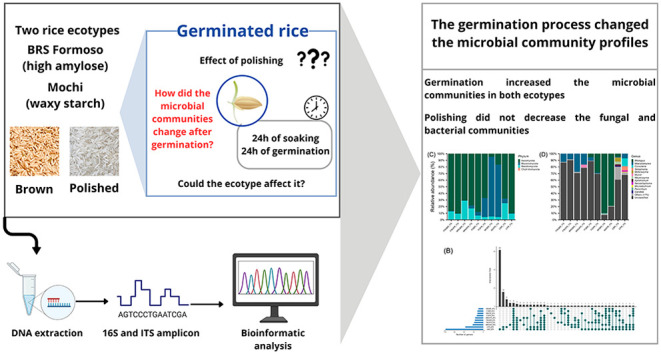

## 1 Introduction

Rice (*Oryza sativa* L.) is an important source of carbohydrates, fibers, vitamins, minerals, and bioactive compounds.^1^ Global rice production is expected to grow by 58 MT to reach 567 MT by 2030, especially in India, China, Vietnam, and Thailand.^2^ *Indica* and *Japonica* are the two major traded varieties of rice on the global market. Those rice varieties can be distinguished by both the shape and amylose concentration. *Indica* has longer grains and more amylose concentration than *Japonica*.^3^ Brown rice (BR) contains all the parts of rice grains (endosperm, embryo, and bran layers) whereas the polished rice (PR) lost those parts after the milling process.^4^ From a nutritional perspective, BR is superior to PR, but some characteristics, such as taste, sensory perception, and shorter shelf life difficult its consumption. New processes such as germination are needed as strategies to help market and improve the sensory quality of BR to increase its consumption.^5^

The natural process of germination could be used to improve the nutritional and sensory quality of rice. During this process, the enzymatic activity increases, causing an increment of a lot of substances, including γ-aminobutyric acid (GABA), phenolic compounds, flavonoids, and γ-oryzanol.^6^ Previous studies have already indeed demonstrated the health benefits of consuming germinated rice on starch digestibility, total antioxidant activities,^7^ and blood pressure.^8^ Consumers search for natural products, free of any harmful ingredients. Despite a massive shift back to the notion of pleasure, pursuing a healthy diet remains a top priority for consumers, who care about what they eat.^9^ In this scenario, germination can be used to improve the sensory characteristics of BR and the nutritional value of PR, as polishing after germination does not cause a significant decrease in bioactive compounds compared to nongerminated rice, which is an advantage since it does not change the consumption of PR, which is preferred by most of the consumers.^10^

Cereal contamination can occur at different stages, including cultivation, harvest, and storage, resulting in diverse microbial communities associated with rice grains.^11^ Germination creates favorable conditions for microbial growth, such as increased moisture, temperature, and the release of nutrients derived from macromolecules degradation. These changes promote an ecological shift in the grain microbiome, often leading to the proliferation of fast-growing bacterial populations and the restructuring of fungal communities.^12^ Previous studies have shown that germination can alter microbial diversity, often increase bacterial abundance while reducing fungal diversity, likely due to competitive interactions and differences in growth dynamics. Thus, germination acts not only as a biochemical process but also as a key ecological driver of microbial succession in cereal grains.^13^

Amplicon-based sequencing approaches have advanced our understanding of food-associated microbiomes, enabling detailed characterization of microbial composition and diversity. In rice, studies have demonstrated that microbial communities shift substantially during germination, with germination acting as a major driver of microbial assembly.^14^ However, most studies have focused on plant development or rhizosphere interactions, with limited attention to postharvest processes. In particular, the combined effects of germination and polishing on both bacterial and fungal communities in rice grains remain poorly explored, particularly across different rice ecotypes. Therefore, this study aimed to investigate the microbial composition and diversity of *Indica* and *Japonica* rice following germination and polishing using an amplicon-based metabarcoding approach.

## 2 Material and Methods

### 2.1 Samples

BRS Formoso (*O. sativa* subsp. *Indica*) (F) and Mochi (M) (*O. sativa* subsp. *Japonica*) were selected from the Active Germplasm Bank of Embrapa *Arroz e Feijão* (Santo Antônio de Goiás- GO, Brazil). The cultivars were chosen according to the apparent amylose content (Table 1) determined according to the method ISO 6647-1.^15^ All genotypes were grown in the 2018/2019 harvest using a flood-irrigated system in the Embrapa Arroz e Feijão experimental field (6°29′8″S, 49°18′32″W). After harvest, the rice grains were naturally dried until they reached 12% humidity.

**Table 1:** Classification, Basic Characteristics, 1000 Grain-Weight and Percentage of Apparent Amylose of BRS Formoso and Mochi Cultivars

	cultivar	
parameters	BRS formoso	mochi
ecotype	*Indica*	*Japonica*
DOI info	10.18730/XCWW4	10.18730/WY19X
biological status	advanced or improved cultivar	semi-natural/sown
1000 grain-weight (g)	30.63	25.46
apparent amylose content (%)	25.60	1.86

### 2.2 Germination Process

Germination was performed according to the methodology described by Zhang et al.^16^ with some modifications. The seeds (500 g) of paddy rice were soaked in deionized water (1 L) at pH 5.6 by adding l-glutamic acid (L-Glu) at 1.0 g/L (Sigma-Aldrich, ref. RES5063G- A701X, St. Louis, USA) and gibberellic acid (GA3) (Sigma-Aldrich, ref. G7645- 5G, St. Louis, USA) at 0.25 mg/L for 24 h in a fan oven (Fabbe-Primar, São Paulo-SP, Brazil) at 30 °C. After this step, the grains were drained and allowed to germinate in a bread fermentation cabinet (National Mfg. Co., Lincoln, USA) at a controlled temperature of 35 °C and relative humidity of 95 % for 24 h. The germinated paddy rice grains were dried in a circulated air oven (Macanuda, Joinville-SC, Brazil) at 50 °C overnight, then husk and pericarp (10 %) were removed with the help of a rice polisher machine Suzuki (Santa Cruz do Rio Pardo- SP, Brazil) for 2 min and subsequently ground in an lab hammer mill 3100 (Perten Instruments AB, Huddinge, Sweden) fit with a 0.8 mm sieve aperture, obtaining a flour that was stored at room temperature until further analyses. The samples evaluated in this study are described in Table 2.

**Table 2:** Description of Rice Samples

sample	cultivar	germination status	processing
FNGBR	BRS formoso	nongerminated	brown rice
FNGPR	BRS formoso	nongerminated	polished rice
FGBR	BRS formoso	germinated	brown rice
FGPR	BRS formoso	germinated	polished rice
MNGBR	mochi	nongerminated	brown rice
MNGPR	mochi	nongerminated	polished rice
MGBR	mochi	germinated	brown rice
MGPR	mochi	germinated	polished rice
CBR	commercial^a^	nongerminated	brown rice
CPR	commercial^a^	nongerminated	polished rice

a Note: commercial samples were used as control and bought in the Rio de Janeiro market.

### 2.3 Extraction of DNA, Amplification of 16S and ITS Regions, and High-Performance Sequencing

Microbial DNA was extracted using DNeasy PowerSoil Pro Kits from QIAGEN (Qiagen, Hilden, Germany), following the manufacturer’s instructions. DNA quality control and library validation were made through High Sensitivity D1000 (Agilent, Santa Clara, California, United States), and metagenomic DNA samples were prepared according to 16S Metagenomic Sequencing Library and Fungal Metagenomic Sequencing Library according to the guidelines for 16S and ITS genes (Illumina, Albany, New York, USA), and subsequently sequenced using an Illumina MiSeq plataform (San Diego, California, USA). For each combination of ecotype × germination × polishing (eight experimental groups plus two commercial controls), the samples were processed and analyzed in duplicate as technical sequencing replicates.

### 2.4 Bioinformatic Processing

ITS primer sequences were removed using Cutadapt^17^ by combining 8 forward and 7 reverse primers (56 total combinations), and processed reads were consolidated into paired FASTQ files. Amplicon data (16S rRNA and ITS) were analyzed using a custom Python-based pipeline (available at https://github.com/LBMCF/pipeline-for-amplicon-analysis), supporting ASV-based analysis. Quality control was performed using FastQC,^18^ followed by read merging, and filtering of low-quality reads with VSEARCH^19^ (maxEE = 0.8; minimum length of 350 bp for bacteria and 200 bp for fungi). Sequence dereplication was conducted with VSEARCH, ASVs were inferred using USEARCH^20^ (--unoise3), and abundance tables were generated with VSEARCH (id = 0.99). Taxonomic classification was assigned using the SINTAX algorithm with SILVA SSU NR v138.2 ^21^ for bacteria and UNITE v10.0 ^22^ for fungi databases.

Alpha-diversity indices (Shannon and Evenness) were calculated using the vegan v2.7.2 package,^23^ with visualization in ggplot2 v4.0.0.^24^ Unclassified taxa were removed from the abundance matrices prior to downstream analyses. For beta diversity, data were Hellinger-transformed and Bray–Curtis dissimilarities were computed using *vegdist* (vegan), followed by ordination through PCoA using *cmdscale*, with graphical representation in ggplot2 and ggrepel v0.9.6 ^25^ packages. Taxonomic profiles at the phylum and genus levels were visualized in ggplot2, with low-abundance taxa grouped as “Others” and unassigned taxa labeled as “Unclassified.” Shared taxa among groups were identified using presence/absence matrices and visualized with UpSetR v1.4.0 ^26^ package. Rarefaction curves were generated using the rarecurve function from vegan, adopting the minimum sequencing depth across samples. To assess the effects of germination, polishing, and ecotype on microbial community structure, permutational multivariate analysis of variance (PERMANOVA) was conducted using adonis2 (vegan) with 9999 permutations, followed by pairwise comparisons using pairwise.adonis v0.4.1 ^27^ with Bonferroni correction.

## 3 Results and Discussion

### 3.1 Germination Increases Bacterial Diversity and Reduces Fungal Diversity

In this study, we employed an amplicon sequencing approach targeting 16S rRNA and ITS regions to examine the microbial diversity and shifts in dominant genera of *Indica* and *Japonica* rice following germination and polishing processes. The rarefaction curves for both data sets approached saturation, indicating adequate sequencing depth and that the observed number of amplicon sequence variants (ASVs) reliably reflected the microbial richness of the rice samples. The alpha diversity indices obtained for the bacterial and fungal communities are presented in Tables S1 and S2 and illustrated in Figures 1 and 2. As each group is represented by a single sample, statistical comparisons between groups for alpha diversity indices were not performed, and results should be interpreted descriptively. Differences in diversity patterns were observed between treatments and among the analyzed ecotypes after germination process (Table S3).

**Figure 1:**
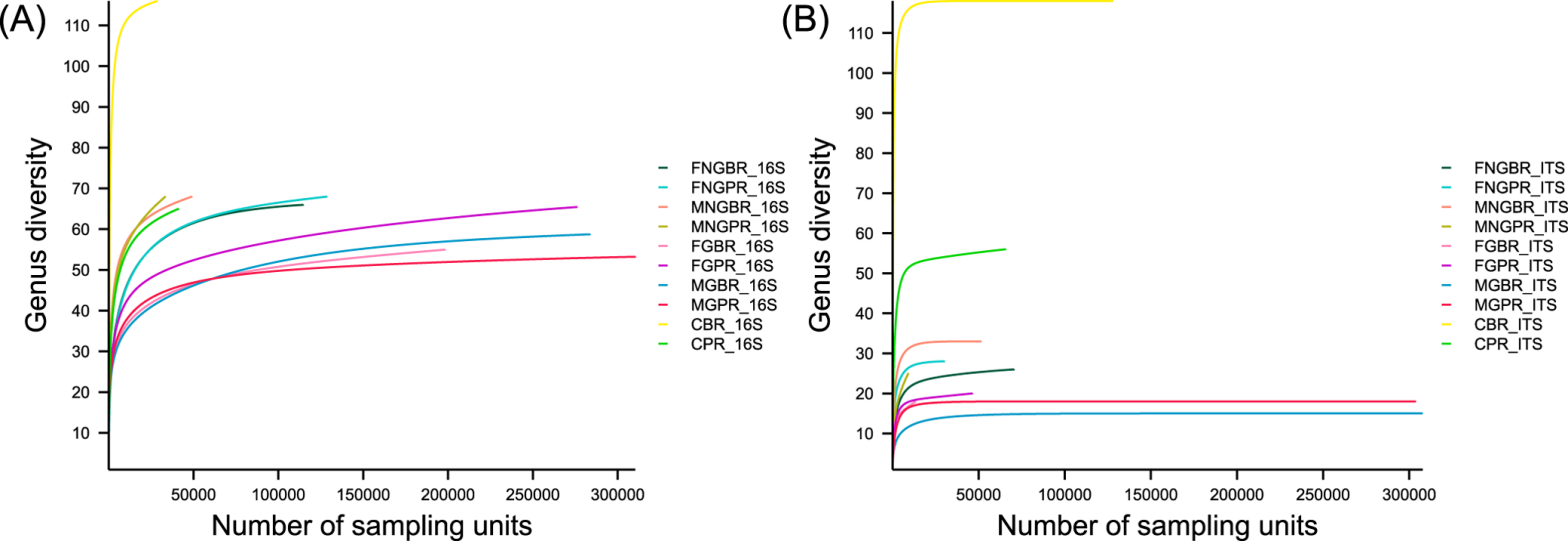
Rarefaction curves of (A) bacterial and (B) fungal sequences of DNA from rice before and after germination and polishing.

**Figure 2:**
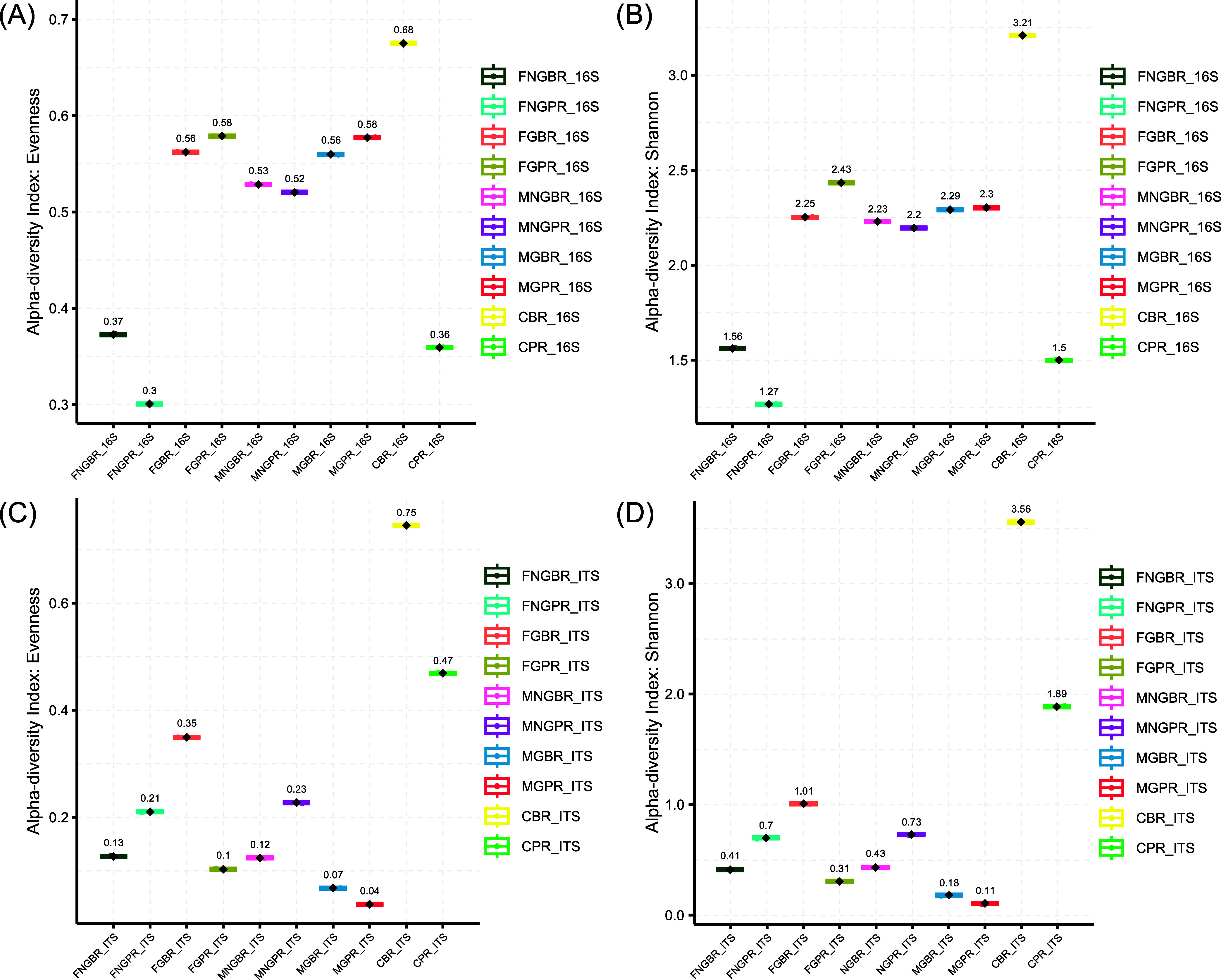
Alpha diversity indices of bacteria and fungi at the genus level (A,C) Evenness (B,D) Shannon index.

For the bacterial community, germination increased the Shannon and Evenness indices compared to the nongerminated samples. The highest Shannon values were observed in CBR (3.21) and FGPR (2.43), followed by FGBR (2.25) and MGBR 2.29) samples. Conversely, the lowest diversities were detected in CPR (1.5) and FNGPR (1.27). These results suggest that the germination process promoted bacterial proliferation and diversification, likely in response to increased moisture, nutrient release, and heightened enzymatic activity during grain sprouting. The higher Evenness values obtained for the germinated samples (0.56–0.58) relative to the nongerminated ones (0.30–0.37) further support that germination favored a more balanced distribution of the microbial community.

In contrast, the fungal community diversity pattern differed substantially from that observed for bacteria. The Shannon index decreased sharply after germination, particularly for the Mochi cultivar, with values dropping from 0.73 (MNGPR) and 0.43 (MNGBR) to only 0.18 (MGBR) and 0.10 (MGPR). A similar, though less pronounced, trend was also observed in the Formoso samples. The highest fungal diversities occurred in the commercial brown (CBR = 3.55) and polished (CPR = 1.89) rice, while the lowest indices were recorded in the germinated samples. The Evenness index exhibited the same behavior, suggesting that the fungal communities became dominated by few taxa after germination. This behavior was also observed by Yang et al.,^30^ in a study assessing the microbial community diversity of 13 samples of germinated brown *Japonica* and *Indica* rice cultivars from China, concluding that bacterial and fungal alpha diversity peaked in nongerminated samples but decreased significantly after grain soaking and germination. This factor may also be associated with the proliferation of bacteria (*Enterobacteriaceae*) and fungi (*Aspergillaceae* or *Coniothyriaceae*). These microbial families are strongly associated with specific recurrent genera that, due to their dominance, may disrupt the microbial balance and consequently reduce community diversity.^29^

Overall, our results indicate an opposite ecological response between the bacterial and fungal communities to germination. While the process creates a microenvironment favorable for bacterial growth, characterized by elevated water activity, temperature, and nutrient mobilization, it simultaneously restricts fungal diversity, possibly due to interspecific competition and oxygen limitation.^30^ Under these conditions, the generation time of bacterial cells may be a significant competitive factor against fungal reproduction, which explains the less abundant results demonstrated by the detected fungal groups.^31,32^

The microbial shifts observed during germination can be explained by a combination of physicochemical changes and ecological selection processes occurring in the grain microenvironment. The imbibition of water and the consequent increase in water activity create favorable conditions for microbial proliferation, while the activation of endogenous enzymes promotes the hydrolysis of macromolecules, leading to the release of simple sugars, amino acids, and other nutrients that support microbial growth. These conditions result in a marked increase in microbial populations during germination, including bacteria, yeasts, and molds, as reported in germinated brown rice systems.^28^

In parallel, germination imposes a selective pressure on the original seed microbiota, favoring microorganisms that are better adapted to hydrated and nutrient-rich environments, while reducing those associated with dry storage conditions.^28,33^ This process is commonly associated with the enrichment of fast-growing and metabolically versatile bacterial genera, such as *Pantoea* and *Bacillus*, alongside shifts in fungal communities, including the proliferation of opportunistic genera such as *Rhizopus*, which are well adapted to high-moisture environments.^28^

Moreover, these changes reflect competitive interactions within the microbial community, where differences in growth rates and metabolic efficiency contribute to the dominance of specific taxa and the reduction in overall diversity.^33^ Additionally, the seed-associated microbiota may be further reshaped according to its functional potential, as microorganisms involved in carbohydrate metabolism and nutrient assimilation tend to be favored during germination.^34^ Together, these mechanisms demonstrate that germination acts as a strong ecological driver, actively restructuring the microbial community through nutrient availability, environmental shifts, and microbial selection processes.

This behavior is further evidenced by Yang et al.,^30^ who observed a decrease in microbial richness and diversity during germination, suggesting that a subset of bacteria or fungi became dominant in the BR samples. This ecological shift indicates that germination can substantially remodel the microbial balance, promoting bacterial dominance and suppressing fungal taxa, demonstrating that competition between these taxonomic groups is inevitable, and the persistence of a specific group will depend mainly on intrinsic and extrinsic factors influenced by the food matrix itself and processing parameters, such as germination and polishing.^35^ Furthermore, the magnitude of these changes appears to be ecotype-dependent: the high-amylose BRS Formoso maintained a relatively higher microbial richness after germination compared to the low-amylose Mochi, suggesting that grain composition drive microbial adaptation and persistence during the germination process. This pattern observed during germination can be explained by the higher water activity in the rice grain, which may favor microbial growth.^28^

### 3.2 Germination Treatment was the Main Factor Associated with Microbial Community Structure

The beta diversity analysis, represented by the PCoA (Figure 4), illustrates differences and similarities in the composition of microbial communities among samples following germination and polishing processes. The observed patterns suggest that germination may be the primary factor influencing community structuring, while polishing appears to have a secondary effect. However, given that each group is represented by a single sample, these observations should be interpreted as exploratory.

**Figure 3:**
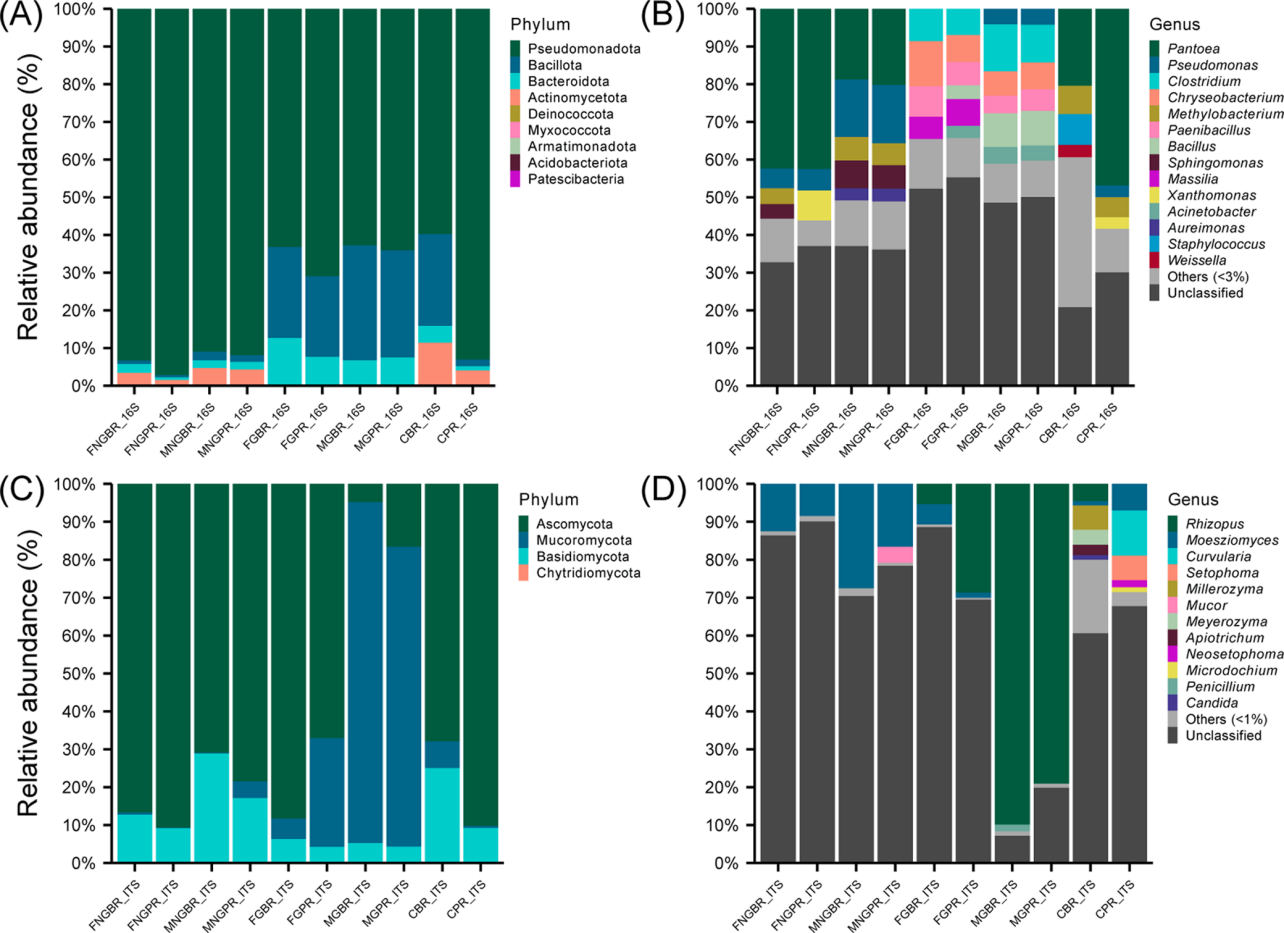
Taxonomic profiles of dominant bacterial (A,B) and fungal (C,D) communities at the phylum and genus level, respectively.

**Figure 4:**
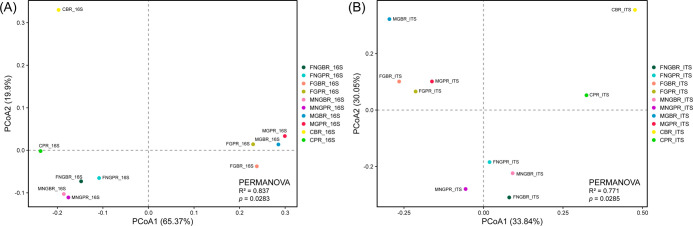
Principal coordinate analysis (PCoA) representations of beta-diversity based on (A) bacterial and (B) fungal of rice samples.

For the bacterial communities, a clear clustering of the germinated samples was observed, independently of the ecotype (FGBR, FGPR, MGBR, and MGPR), indicating that germination similarly modulated the bacterial composition in both rice types (Figure 4). A similar pattern of bacterial modulation was also reported by Yang et al.,^28^ who observed convergence of microbial communities during the germination of brown rice germination, where bacterial diversity peaked in nongerminated samples and decreased significantly after soaking and germination. This behavior suggests that the conditions observed during the germination, such as increased moisture, temperature, and nutrient release, created an ecological microenvironment that favored similar microbial groups, reducing the differences between the *Indica* (BRS Formoso) and *Japonica* (Mochi) cultivars. Conversely, the nongerminated samples (FNGBR, FNGPR, MNGBR, and MNGPR), as well as the commercial controls (CBR and CPR), formed closer clusters, reflecting less diverse microbial communities with equitability formed closer clusters, reflecting less diversified and more homogeneous microbial communities, likely derived from the grain’s natural microbiota and storage. Similar results were observed by Nanfack et al.,^36^ who reported distinct clustering patterns associated with rice seed germination performance, reinforcing the role of the process on microbial structure.

In contrast, the fungal communities displayed greater dispersion along the PCoA axes, demonstrating a more heterogeneous behavior among the treatments. Although some groupings were observed, such as the pairing of FNGBR and FNGPR, or the proximity between FGPR and FGBR, the germinated Mochi rice samples (MGBR and MGPR) showed more pronounced separation, possibly associated with the dominance of *Mucoromycota* (fungi) identified under these conditions. This differentiation may reflect a more sensitive fungal response to the physicochemical variations induced by germination, such as changes in pH, oxygenation, and substrate availability. Similarly, Wang et al.^37^ observed that moisture variations during rice storage strongly influence fungal succession and community heterogeneity, which corroborates the pattern identified in this study.

Overall, the beta diversity findings reinforce that germination plays a central role in the restructuring of the rice microbiota, promoting convergence in bacterial communities and greater variability in fungal communities. This behavior indicates that bacteria exhibit a high capacity for adaptation and colonization under the conditions imposed by the germination process, which aligns with Dhondge et al.,^38^ who observed a high adaptive capacity of bacterial communities in rice rhizosphere environments. Conversely, fungi appeared more susceptible to environmental fluctuations, who suggested high sensitivity of cereal fungal communities to variations in moisture and temperature.^39^

The relative homogenization of the germinated samples also suggests that germination, as opposed to genotype, is the main factor driving microbial composition. This finding is consistent with observations by Jang et al.,^40^ who reported that environmental factors exert a more pronounced influence on the microbial structure of rice than genetic differences between plant cultivars. Thus, the findings of our study indicate that the impact of germination on the microbial structure of rice is more relevant than the effect of polishing or plant ecotype. These characteristics are also corroborated by Yang et al.,^28^ who showed that the germination process is more impactful than polishing on the variation and diversity of the microbiome in BR rice samples. Therefore, the germination process significantly modulated the microbial composition and reduced the microbial diversity of the analyzed samples.

To complement the analysis of community composition, PERMANOVA was applied to assess differences between the predefined groups. Differences were observed between germinated and non-germinated samples in both bacterial and fungal data sets, with relatively high effect sizes (16S rRNA: *R* ^2^ = 0.84, *p* = 0.0283; ITS: *R* ^2^ = 0.77, p = 0.0285). In contrast, comparisons involving brown vs polished rice and *Indica* vs *Japonica* ecotypes resulted in low *R* ^2^ values and nonsignificant *p*-values (Table S3). However, these results should be interpreted with caution due to the absence of biological replicates, which limits the statistical power and generalization of the findings.

### 3.3 Germination Promotes the Dominance of Specific Bacterial Genera and Reshapes Fungal Communities

#### 3.3.1 Phylum Level Distribution

The taxonomic analysis revealed marked differences in the composition of bacterial and fungal communities between treatments and ecotypes, especially after the germination process. The microbial profile of the BRS Formoso, Mochi, and commercial rice samples showed the dominance of the phyla *Pseudomonadota* and Ascomycota for Bacteria and Fungi, respectively. Other bacterial groups detected also included *Bacillota*, *Bacteroidota*, *Actinomycetota*, *Deionococcota*, *Myxococcota*, *Armatimonadota*, *Acidobacteriota*, and *Patescibacteri*a (Figure 3A). The bacterial communities were dominated mainly by *Pseudomonadota*, followed by *Bacillota*, *Actinomycetota*, and *Bacteroidota*. This taxonomic composition is consistent with previous studies on the rice microbiome, which point to *Pseudomonadota* (Proteobacteria) as the dominant phylum in seeds, grains, and plant tissues, regardless of geographical origin or genotype.^40–42^

In the non-germinated samples, the dominance of *Pseudomonadota* (Proteobacteria) was particularly evident, especially in the polished samples (FNGPR and MNGPR), suggesting a more restricted microbial community adapted to conditions of low moisture and substrate availability. Conversely, the germination process promoted a relative increase in *Bacillota*, particularly in the germinated samples of both ecotypes (FGBR, FGPR, MGBR, and MGPR). This behavior aligns with observations from recent studies, which reported the expansion of *Bacillota* during germination or initial rice sprout development, possibly associated with the release of simple organic compounds and higher oxygen availability.^43^ These groups include taxa such as *Bacillus* and *Paenibacillus*, widely recognized for their ability to form spores, resist variable conditions, and also act as plant growth-promoting microorganisms.

Furthermore, a proportional increase in *Actinobacteriota* was observed in the germinated samples, suggesting that these microorganisms play a role in the degradation of structural compounds released by the hydrolysis of starch and proteins in the endosperm. This pattern has been previously reported in metagenomic analyses of rice and its rhizosphere, indicating the involvement of Actinobacteria in polymer decomposition and antimicrobial metabolite production.^44,45^ In contrast, the relative abundance of *Bacteroidota* was lower in the germinated and polished samples, possibly reflecting the sensitivity of this group to the environmental changes imposed by germination.

Regarding the fungal community, the structure at the phylum level was dominated by *Ascomycota*, *Basidiomycota*, and, to a lesser extent, *Mucoromycota*. Ascomycota dominated in the non-germinated and commercial samples (CBR, CPR), reflecting the presence of mycelial fungi typical of stored grains, such as *Aspergillus* and *Penicillium*. Studies on rice storage show a similar pattern, with Ascomycota representing between 60% and 80% of the detected sequences.^36,46^ Following germination; however, a reduction in Ascomycota and a relative increase in *Mucoromycota* were observed, especially in the germinated Mochi cultivar samples. This ecological substitution is characteristic of humid, nutrient-rich environments, where fast-growing opportunistic fungi, such as *Mucor*, replace typical storage-associated saprophytic species.^46^

Nevertheless, Basidiomycota showed minor participation across all treatments, with only a slight increase in the germinated brown rice samples. This group includes fungi that can act in the decomposition of phenolic and lignocellulosic compounds.^47^ Thus, the balance between *Ascomycota*a and Mucoromycota may be a consequence of the ecological reorganization resulting from germination, where hydration and metabolic activation of the grains shift the fungal community structure toward a more opportunistic taxa adapted to a more humid environments, while reducing the abundance of storage-associated fungi.^38^ Generally, the phylum-level analysis demonstrates that the germination process is the main driver for remodeling the rice microbial community, promoting the replacement of dominant groups adapted to dry storage with microorganisms exhibiting higher metabolic activity and colonization capacity.

#### 3.3.2 Genus Level Distribution

The analysis of microbial communities at the genus level evidenced substantial changes in the composition and relative abundance of the main bacterial and fungal taxa following the germination and polishing processes (Figure 3BD). Among bacteria, the most abundant genera in the non-germinated samples were *Pantoea*, *Enterobacter*, *Sphingomonas*, and *Pseudomonas*, predominantly belonging to the phylum *Pseudomonadota* (Proteobacteria). These genera are widely reported as components of the seed and grain rice microbiome, often associated with the natural epiphytic and endophytic microbiota.^40,41^ Their dominance in the non-germinated and commercial samples (CBR, CPR) indicates that these communities largely reflect the microbiome inherited from the seed and the storage environment. *Pantoea* and *Enterobacter* are known to tolerate low moisture levels and resist oxidative stress conditions, which explains their dominance in dry and polished samples.^48^

Following germination, a pronounced shift in bacterial community structure was observed, with the relative increase of genera from the phylum *Bacillota*, mainly *Bacillus*, *Paenibacillus*, and *Lysinibacillus*, in addition to representatives of *Actinobacteriota*, such as *Curtobacterium* and *Microbacterium*. These genera are typically associated with the rhizosphere and the interior of germinating seedlings, playing important roles in nutrient solubilization, phytohormone production, and pathogen protection.^42,43^ The abundant presence of *Bacillus* in the germinated samples (FGBR, FGPR, MGBR, and MGPR) aligns with studies demonstrating that genus ability to proliferate during germination due to increased enzymatic activity and the availability of organic substrates.^45^

This replacement of *Pantoea* and *Enterobacter* by *Bacillus* and *Paenibacillus* represents an ecological transition typical of environments shifting from low water activity to high moisture and active metabolism conditions, as occurs during germination. Furthermore, differences between the plant ecotypes suggest an effect of starch type and grain structure. BRS Formoso maintained higher diversity of bacterial genera after germination, while Mochi, with lower amylose content, showed communities more dominated by *Bacillus* and *Paenibacillus*. This trend may be related to the higher availability of simple sugars and nitrogenous compounds released in waxy grains, which favors the rapid growth of opportunistic bacteria.^28,45^

Fungal communities also exhibited significant changes after germination (Figure 3C). In the non-germinated and commercial samples, the genera *Aspergillus*, *Penicillium*, *Cladosporium*, and *Alternaria* were dominant, all belonging to the phylum Ascomycota. These fungi are characteristic of stored grains, desiccation-tolerant, and able of colonizing starch-rich surfaces under low moisture.^46^ Nonetheless, after germination, the abundance of these genera substantially decreased, with a high increase of *Mucor* and *Rhizopus* (*Mucoromycota*), especially in the germinated Mochi cultivar samples. These fungi are fast-growing and easily colonize moist, nutrient-rich substrates on simple carbohydrates, which is consistent with the environment created during the germination process.^36,45^ The replacement of *Aspergillus* and *Penicillium* by *Mucor* and *Rhizopus* reflects an ecological shift driven by the physicochemical conditions of germination, in which water and oxygen availability favors species with rapid metabolism and lower stress resistance. Furthermore, the presence of *Cladosporium* and *Alternaria* in some germinated samples, albeit in lower abundance, may indicate remnant microorganisms from the original microbiome, possibly persistent on the grain surface.^49^

Generally, the genus-level analyses demonstrate that germination acts as a microbial community reorganization factor, promoting the growth of beneficial bacterial taxa, such as *Bacillus* and *Paenibacillus*, and reducing the diversity of typical storage saprophytic fungi (Figure 5). The polishing process, in turn, exerted a less pronounced effect, but contributed to a reduction in total diversity by removing the outer layers of the grain where many microorganisms are concentrated. This reorganization suggests that germination, more than the plant cultivar type, defines the final structure of the rice-associated microbiota, favoring communities adapted to metabolic activities such as starch and protein hydrolysis, release of sugars and amino acids, and increased respiration and oxygenation.^42,47^

**Figure 5:**
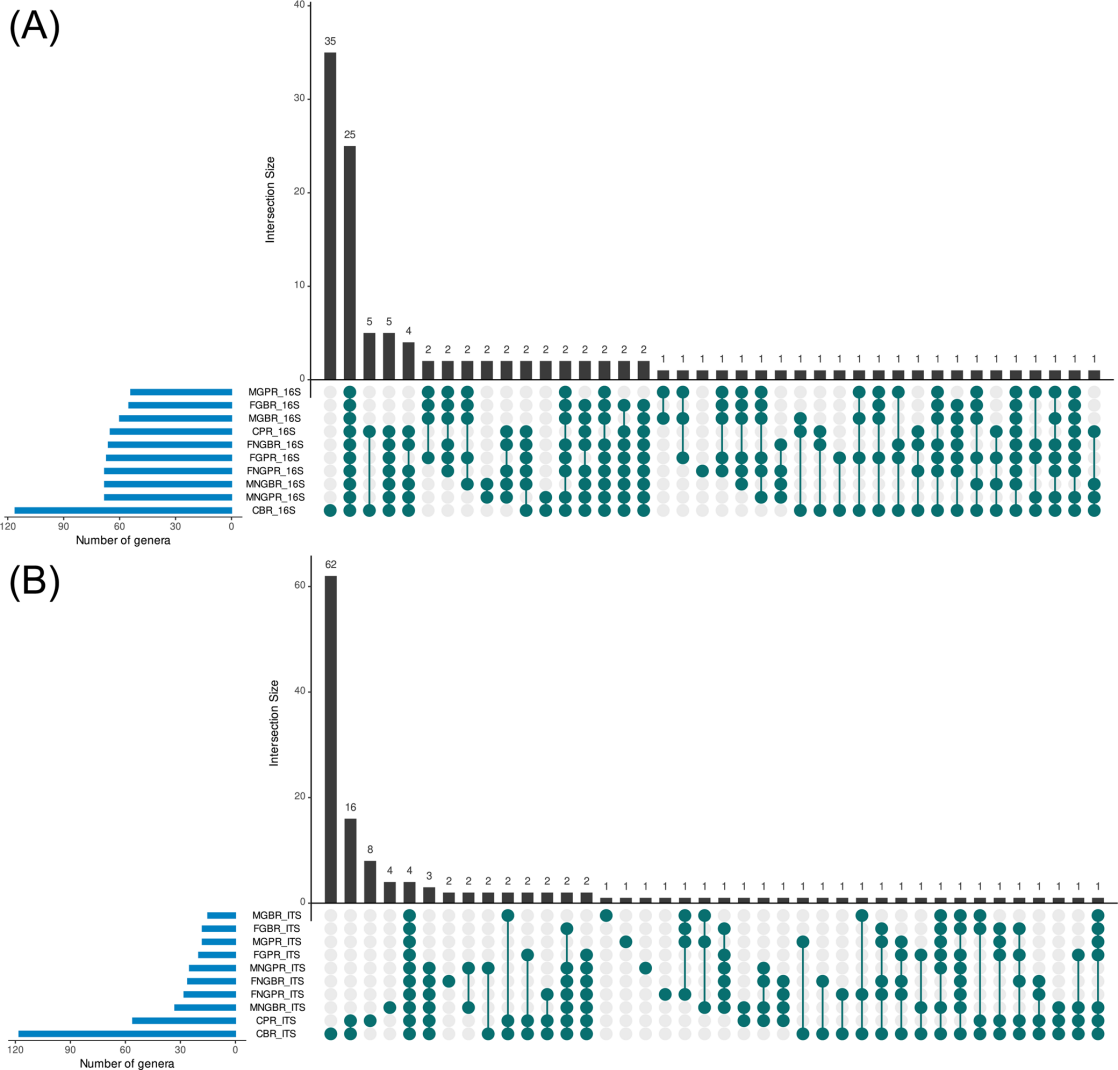
UpSet graph showing the number of shared genera of bacteria (A) and fungi (B).

### 3.4 Germination Drives Microbial Succession and Ecological Restructuring in Rice Grains

The integrated analysis of the results obtained in the previous sections suggests that the germination treatment employed in this study was the main factor associated with the reorganization of microbial communities associated with rice grains, superseding the effects of polishing and differences between plant ecotypes. This restructuring involves a striking microbial succession, with the replacement of groups associated with dry storage by microorganisms adapted to conditions of higher moisture, active metabolism, and the release of organic substrates.^28,45^

In addition to the compositional and diversity patterns described above, the multivariate PERMANOVA analysis supported these observations by showing that only the germination contrast resulted in statistically significant differences in overall community structure for both bacterial and fungal data sets. Conversely, the comparisons involving brown vs polished rice and *Indica* vs *Japonica* ecotypes did not yield significant differences, indicating that these factors contributed minimally to the total variation detected in this study.

The dominance of *Pseudomonadota* (Proteobacteria) in the non-germinated samples gave way to phyla such as *Bacillota* and *Actinobacteriota* after germination, notably the genera *Bacillus*, *Paenibacillus*, and *Curtobacterium*. This transition is linked to the greater availability of simple sugars and amino acids released by the hydrolysis of macromolecules during the germination process.^45^ Moreover, recent studies show that germinating rice seeds release volatile compounds like benzaldehyde, capable of selectively recruiting rhizospheric bacteria and remodeling the surrounding microbial structure.^50^ This phenomenon helps explain the relative increase of the abundance of *Bacillus* and *Paenibacillus* observed in the germinated samples.

In the fungal communities, the same pattern of succession was evident, in which the typical storage fungi, such as *Aspergillus*, *Penicillium*, and *Cladosporium*, decreased sharply after germination, while fast-growing species of the genera *Mucor* and *Rhizopus* became dominant.^46^ This substitution may be directly associated with the increase in water activity and the availability of fermentable substrates, creating an environment more favorable for opportunistic fungi and less conducive to xerophilic species. Similar patterns have been observed in other germinated cereals, such as wheat and barley, reinforcing that fungal succession is a universal ecological response to the germination process.^36^

Polishing also contributed to modifying the microbial structure, albeit more subtly. Studies demonstrate that the outer layers of the grain harbor a large proportion of endophytic and saprophytic microorganisms, whose removal reduces total diversity and microbial load.^47^ This explains the lower richness observed in the polished samples compared to the brown ones, especially in the Mochi cultivar.

The differences observed between the BRS Formoso and Mochi ecotypes indicate that genetic and structural grain factors, such as amylose content and the composition of pericarp layers, modulate the final microbial composition.^30,51^ Waxy rice Mochi, with lower amylose content, showed communities more dominated by *Bacillus* and *Mucor*, while Formoso maintained higher bacterial and fungal diversity. Such differences are consistent with ecological patterns described between wild and cultivated rice species, where domestication reduced microbial diversity and selected specific functional groups adapted to the cultivation environment.^30^

From a functional perspective, the microbial reorganization observed during germination has important ecological and technological implications. Ecologically, the process simplifies the community structure and selects taxa with greater competitive ability, production of hydrolytic enzymes, and resistance to environmental fluctuations. Technologically, this change can impact the quality and safety of germinated rice, reducing the presence of mycotoxin-producing fungi (*Aspergillus*, *Penicillium*) and favoring the dominance of potentially beneficial bacteria, such as *Bacillus*, associated with microbiological stability and the production of antimicrobial metabolites.^45,50^ Therefore, our results suggest that germination redefines the interactions between bacteria and fungi in the grain microenvironment, promoting a more active microbial system adapted to high moisture and metabolic activity. This community reorganization is closely linked to the physicochemical changes occurring during germination and highlights the relevance of these microbial shifts for food quality and safety, as well as their implications for postharvest processing strategies.^30^

This study expands current knowledge on rice-associated microbiomes by integrating the effects of germination and polishing across distinct rice ecotypes, an approach that remains limited in previous studies. While earlier research has primarily focused on microbial dynamics during germination or on seed-to-seedling transitions under controlled conditions,^28,34^ our findings demonstrate that postharvest processing steps can play a decisive role in shaping microbial community structure.

In particular, the consistent convergence of bacterial communities across ecotypes following germination, combined with the relatively minor influence of polishing, provides new evidence that environmental and processing factors may override intrinsic grain characteristics in determining microbiome composition.^30^ These results complement previous studies while offering a more comprehensive perspective by simultaneously evaluating bacterial and fungal communities within a food-processing context, thereby contributing to a better understanding of the ecological and technological implications of rice microbiome modulation.^52^

In summary, the germination treatment employed in this study was identified as the main factor associated with microbial community restructuring in rice under the conditions evaluated in this study, surpassing the effects of polishing and plant ecotype. This process promoted a clear microbial succession, characterized by an increase in bacterial diversity and the replacement of storage-associated taxa, such as *Pantoea* and *Enterobacter*, by genera commonly associated with active metabolism and ecological competitiveness, including *Bacillus*, *Paenibacillus*, and *Curtobacterium*. From a food safety perspective, this shift is particularly relevant, as germination markedly reduced the relative abundance of filamentous fungi typically linked to grain storage and mycotoxin production, such as *Aspergillus* and *Penicillium*, while favoring fast-growing bacterial communities adapted to high water activity. Conversely, the dominance of bacterial taxa with high metabolic activity under germination conditions highlights the need for careful control of processing parameters, as these microorganisms may influence microbial stability during postgermination handling, drying, storage, and further food preparation. The reduction in fungal diversity observed in this study, together with the increased relative abundance of bacterial communities, suggests that germination may act as a microbial selection step under the evaluated conditions, potentially improving microbiological safety when adequately managed, but also introducing new ecological dynamics that require monitoring. In addition, it should be noted that the germination protocol included supplementation with l-glutamic acid and GA3. Therefore, the microbial shifts observed in this study reflect the combined effects of germination and additive supplementation. Future studies including germination controls without these additives are needed to disentangle their individual contributions to microbiome restructuring. Overall, these findings demonstrate that germination reshapes the rice microbiome in ways that are relevant to food quality and safety; however, it is important to note that this study was based on a limited number of samples without biological replicates, and further studies including larger sample sizes, experimental replicates, and time-resolved sampling are necessary to validate these trends and support the development of optimized germination strategies aimed at promoting beneficial microorganisms.

## Supplementary Material



## Data Availability

Raw and processed data is available through SRA database (NCBI BioProject ID PRJNA1089529).

## Notes

Abundance and diversity indexes of bacterial populations (Table S1); Abundance and diversity indexes of fungal populations (Table S2); Results for analysis of community composition (PERMANOVA) (Table S3) (PDF)

## Abbreviations

ASV: amplicon sequence variant
BR: brown rice
DNA: deoxyribonucleic acid
GABA: γ-aminobutyric acid
GA3: gibberellic acid
GBR: germinated brown rice
ITS: internal transcribed spacer
l-Glu: l-glutamic acid
OTUs: operational taxonomic units
PCoA: principal coordinates analysis
PERMANOVA: permutational multivariate analysis of variance
PR: polished rice
RNA: ribonucleic acid
rRNA: rRNA
16S rRNA: 16S ribosomal ribonucleic acid
