# Supplementary material for: Germination and Polishing Reshape Microbial Communities in Japonica and Indica Rice
Source: J Agric Food Chem. 2026 Jul 8;74(28):22337–47. doi: 10.1021/acs.jafc.6c02819 (PMC13422772; doi:10.1021/acs.jafc.6c02819)
Supplement: Supplementary file 1 [file jf6c02819_si_001.pdf]

Support information

Germination and polishing reshape microbial communities in *Japonica* and *Indica* rice

Maria Eugenia Araujo Silva Oliveira[1]*, Daniel Lucino[2], Glen Jasper Yupanqui Garcia[3], Bruno Gerfi Bertozzi[2], Priscila Zaczuk Bassinello[4], José Manoel Colombari Filho[5], Carlos Wanderlei Piler de Carvalho[6], Aristóteles Góes-Neto[3], Liliana de Oliveira Rocha[2], Dirce Yorika Kabuki[2], Otniel Freitas Silva[1,6], Cristina Yoshie Takeiti[1,6]*

[1]Programa de Pós-Graduação em Alimentos e Nutrição (PPGAN), Universidade Federal do Estado do Rio de Janeiro, UNIRIO, Avenida Pateurs, 296, 22290-240, Rio de Janeiro, RJ, Brasil

[2]Departamento de Ciência de Alimentos e Nutrição (DECAN), Programa de Pós-Graduação em Ciência de Alimentos, Faculdade de Engenharia de Alimentos (FEA), Universidade Estadual de Campinas (UNICAMP), Rua Monteiro Lobato, 80, Cidade Universitária Zeferino Vaz, 13083-862, Campinas, SP, Brasil

[3]Departamento de Microbiologia, Programa de Pós-Graduação em Bioinformática, Instituto de Ciências Biológicas, Universidade Federal de Minas Gerais, UFMG, Avenida Antônio Carlos, 6627, Pampulha, 31270-901, Belo Horizonte, MG, Brasil

[4]Embrapa Alimentos e Territórios, Rua Cincinato Pinto, 348, 57020-050, Maceió, AL, Brasil

[5]Embrapa Arroz e Feijão, Rodovia GO-462, Km 12, Zona Rural, 75375-000, Santo Antônio de Goiás, GO, Brasil

[6]Embrapa Agroindústria de Alimentos, Avenida das Américas, 29501, 23020-470, Rio de Janeiro, RJ, Brasil

*Corresponding author

Table S1. Abundance and diversity indexes of bacterial populations

Table S2. Abundance and diversity indexes of fungal populations

Table S3. Results for analysis of community composition (PERMANOVA)

Table S1. Abundance and diversity indexes of bacterial populations

Sample	Evenness	Shannon
FNGBR	0.373	1.561
FNGPR	0.301	1.268
FGBR	0.563	2.252
FGPR	0.579	2.433
MNGBR	0.529	2.230
MNGPR	0.521	2.196
MGBR	0.560	2.291
MGPR	0.578	2.302
CBR	0.676	3.201
CPR	0.360	1.499

Table S2. Abundance and diversity indexes of fungal populations

Sample	Evenness	Shannon
FNGBR	0.128	0.414
FNGPR	0.211	0.702
FGBR	0.350	1.010
FGPR	0.104	0.309
MNGBR	0.125	0.435
MNGPR	0.230	0.732
MGBR	0.068	0.184
MGPR	0.040	0.109
CBR	0.745	3.556
CPR	0.470	1.888

Table S3. Results for analysis of community composition (PERMANOVA)

Pairs	Method	R2	*p*.value	*p*.adjusted	*p*.permanova
Germinated vs Non-Germinated	16S rRNA	0.837386	0.0283	0.0283	0.0263
Brown vs Polished	16S rRNA	0.01978	0.9042	0.9042	0.906
Indica vs Japonica	16S rRNA	0.074671	0.5108	0.5108	0.5131
Germinated vs Non-germinated	ITS	0.771033	0.0285	0.0285	0.028
Brown vs Polished	ITS	0.029062	0.9633	0.9633	0.9616
Indica vs Japonica	ITS	0.054269	0.5415	0.5415	0.5495
